# Pill-induced esophagitis caused by ingesting excessive caffeine tablets

**DOI:** 10.1007/s12328-019-01055-w

**Published:** 2019-10-23

**Authors:** Jun Miyata, Yoshiyuki Ito, Shigeji Ito

**Affiliations:** Department of Internal Medicine, Tannan Regional Medical Center, 1-2-31 Sanrokucho, Sabae, Fukui 916-8515 Japan

**Keywords:** Pill-induced esophagitis, Caffeine overdose, Gastrointestinal endoscopy, Diffuse ulcer

## Abstract

A 19-year-old woman with suicidal thoughts consumed 24 anhydrous caffeine tablets and was admitted to our hospital. After being discharged from the hospital, her oral intake remained impaired because of retrosternal pain and she was readmitted. An upper gastrointestinal endoscopy revealed diffuse ulcers throughout the mid-to-lower esophagus; the patient was diagnosed with caffeine-induced esophagitis. She recovered soon after conservative treatment. A follow-up endoscopy performed 1 month after the patient was discharged showed that the ulcers had healed. This case highlights the risk of esophageal injuries after ingesting excessive caffeine tablets, which were sold as dietary supplement without a prescription. Our experience indicates that endoscopic surveillance is advisable to prevent severe complications if a patient presents with esophageal symptoms suggestive of chemical esophagitis.

## Introduction

Caffeine is a widely used substance that acts as a mental and physical performance enhancer. Anhydrous caffeine tablets can easily be purchased as dietary supplements, online or at pharmacies, without a prescription. Because caffeine is readily accessible and a caffeine overdose can cause death, the substance is used in suicide attempts. Kamijo et al. analyzed 101 patients from 38 emergency departments in Japan, between April 2011 and March 2016, after ingesting an overdose of caffeine [1]. The authors reported that seven patients experienced cardiac arrest, three of which died. The authors also reported that the number of patients presenting with caffeine intoxication to the 38 emergency departments had greatly increased since 2013.

Caffeine overdose frequently induces upper gastrointestinal symptoms, such as nausea, vomiting and epigastralgia [2-4]. However, the number of patients who suffer from esophageal injuries is extremely low. Hence the pathophysiological and endoscopic characteristics of caffeine-induced esophageal injury remain unclear. Here, we report a case of pill-induced esophagitis caused by ingesting excessive caffeine tablets that was diagnosed and followed by endoscopy.

## Case report

A 19-year-old Japanese woman with suicidal thoughts consumed 24 tablets of Estaron-mocha (SSP Co. Ltd., Japan), which is an over-the-counter drug containing 100 mg of anhydrous caffeine and 5 mg thiamine nitrate per tablet [5]. The woman was brought to the emergency department of our hospital presenting with retrosternal pain, nausea, brown-colored vomit, hand tremors, and slight disorientation after her overdose. In the emergency department the patient’s level of consciousness deteriorated and she became non-ambulatory. She was diagnosed with acute caffeine intoxication and was admitted to the hospital for close observation. The patient was discharged the following day after her level of consciousness improved. However, she returned to our hospital the next day and reported experiencing persistent epigastric and retrosternal pain that markedly impaired her oral intake. The patient was readmitted to the hospital for further evaluation.

A physical examination of the patient showed that she was 158 cm tall and weighed 73 kg (with a body mass index of 29.2 kg/m^2^). Her temperature was 37.6 ºC, her pulse was 67 beats per minute, and her blood pressure was 117/89 mmHg. The patient’s abdomen was soft and non-distended, but she reported tenderness at her epigastric fossa and in her right hypochondrium. The patient’s bowel sounds were normal. All other physical examination findings were normal.

**Fig. 1:**
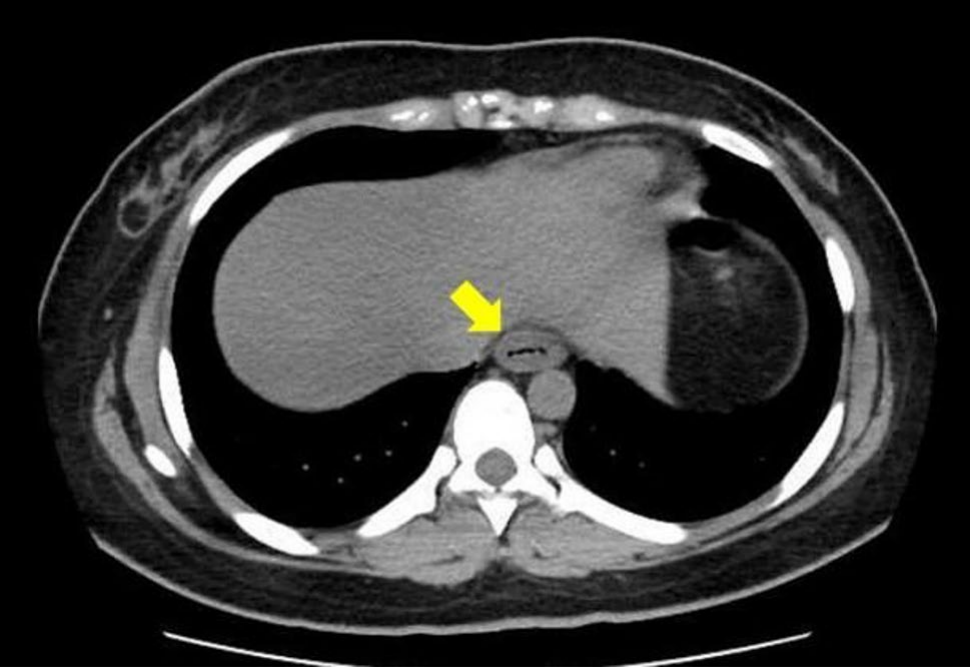
An abdominal axial computed tomography scan revealed concentric esophageal wall thickening (indicated by the arrow)

The patient’s laboratory test results are shown in Table 1. Her blood levels of white blood cells, creatinine phosphokinase, and C-reactive protein were elevated. Unfortunately, the patient’s blood level of caffeine was not assessed. An axial computed tomography scan of the abdomen showed concentric wall thickening in the lower esophagus, most likely representing transmural inflammation (Fig. 1).

**Table 1:** Laboratory data on admission

Complete blood count		Blood chemistry and serology		Urinalysis	
White blood cells	13700 /μL	Total protein	8.4 g/dL	Specific gravity	1.010
Red blood cells	430×10^4^ /μL	Albumin	4.9 g/dL	pH	6.5
Hemoglobin	12.5 g/dL	Total bilirubin	0.76 mg/dL	Protein	2+
Hematocrit	39.5%	AST	37 U/L	Glucose	–
Platelet count	34.9×10^4^ /μL	ALT	31 U/L	Ketone body	–
		LDH	251 U/L	Blood	3+
		ALP	162 U/L	Leukocyte	3+
		γGTP	35 U/L	Urinary protein	63.0 mg/dL
		CPK	372 U/L		
		Amylase	35 U/L		
		BUN	10.2 mg/dL		
		Creatinine	0.81 mg/dL		
		Na	143.0 mEq/L		
		K	3.95 mEq/L		
		Cl	102.9 mEq/L		
		Glucose	104 mg/dL		
		CRP	6.77 mg/dL		

*AST* aspartate aminotransferase, *ALT* alanine aminotransferase, *LDH* lactate dehydrogenase, *ALP* alkaline phosphatase, *γGTP* gamma-glutamyl transferase, *CPK* creatine phosphokinase, *BUN* blood urea nitrogen, *Na* sodium, *K* potassium, *Cl* chloride, *CRP*, C-reactive protein

**Fig. 2:**
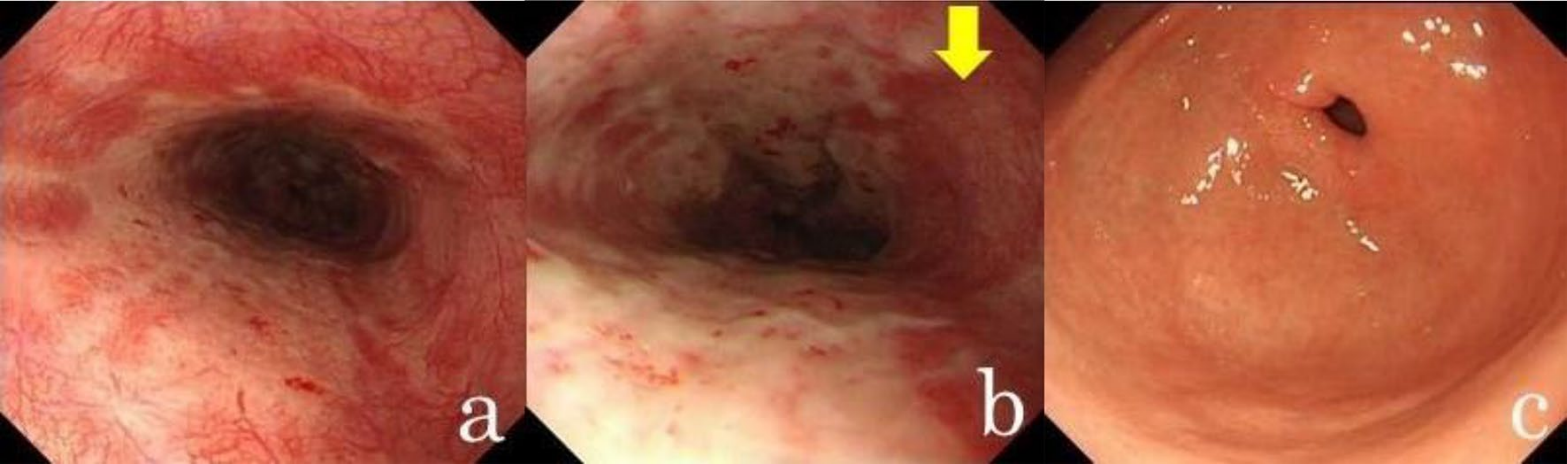
A diagnostic endoscopy revealed diffuse ulcers. **a** Ulcers in the proximal portion of the region (middle esophagus) were circumferential shallow ulcers. **b** Ulcers in the lower esophagus were non-circumferential and were adjacent to the normal mucosa with a submucosal vascular network (indicated by the arrow). **c** No other gastroduodenal lesions were detected

A diagnostic endoscopy performed 4 days after the patient ingested the caffeine tablets revealed diffuse ulcers throughout the mid-to-lower esophagus (Fig. 2). Circumferential, shallow ulcers were observed in the middle esophagus (Fig. 2a); whereas non-circumferential ulcers, adjacent to the normal mucosa with submucosal vascular network, were detected in the lower esophagus (Fig. 2b). A hiatal hernia was not present. No other gastroduodenal lesions were detected (Fig. 2c).

**Fig. 3:**
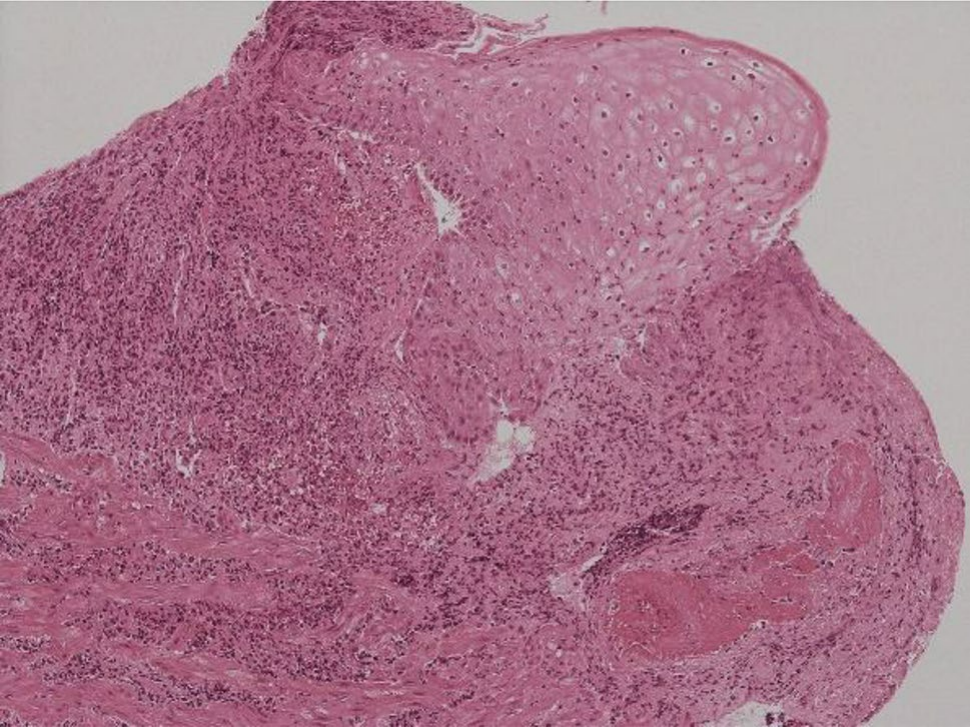
Biopsies of the esophageal lesions showed nonspecific inflammation with marked neutrophil infiltration (Hematoxylin and eosin × 100)

Biopsies were obtained from erosions and showed marked neutrophil infiltration, which indicates nonspecific inflammation. Eosinophil infiltration, malignant cells, specific pathogens, or intranuclear inclusion bodies were not seen (Fig. 3). Based on the patient’s medical history and the endoscopic findings, the patient was diagnosed with pill-induced esophagitis caused by ingesting excessive caffeine tablets.

**Fig. 4:**
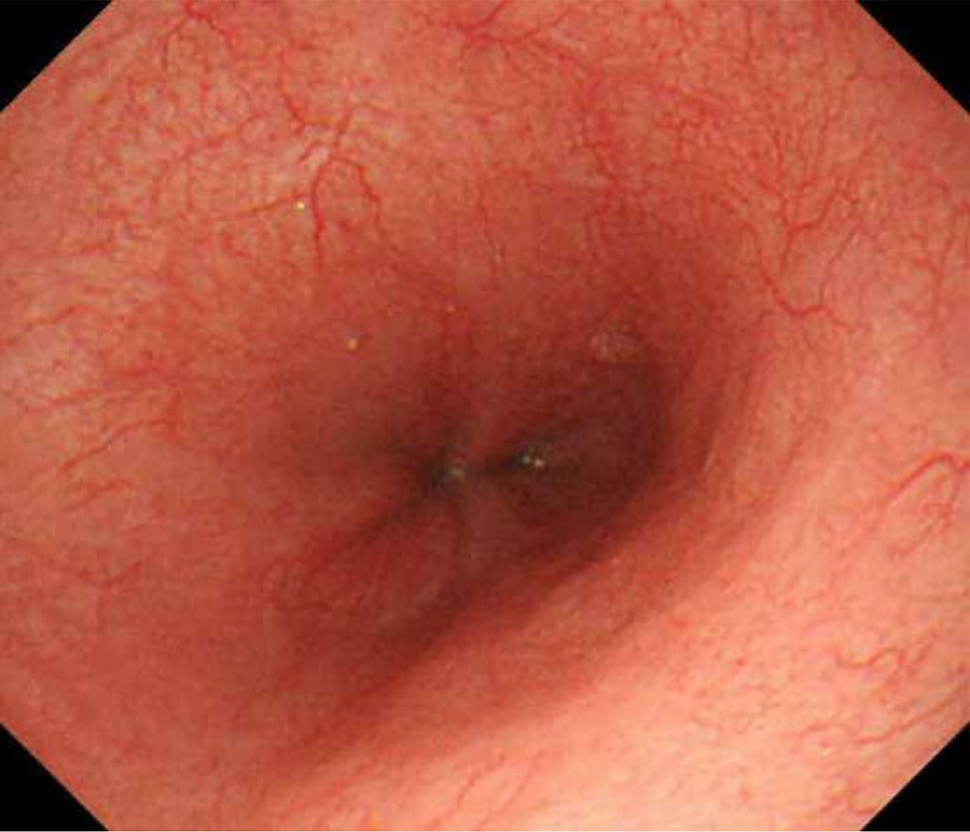
A follow-up endoscopy performed 1 month after the patient was discharged from the hospital showed that the ulcers had resolved

The patient resumed oral intake of a liquid diet soon after the endoscopy was performed. The patient was prescribed oral esomeprazole (20 mg) to be taken once a day after lunch and oral alginic acid (5%, 20 mL) to be taken three times a day before each meal. She gradually recovered and was discharged 8 days after readmission. A follow-up endoscopy performed 1 month after the patient was discharged home showed that the diffuse erosions had healed and that there were no endoscopic features suggesting gastroesophageal reflux disease (Fig. 4).

## Discussion

Pill-induced esophagitis refers to mucosal injury caused by a lodged tablet in the esophageal lumen. A variety of drugs have been reported to induce esophagitis, and the drugs most frequently implicated are antibiotics, bisphosphonates, and non-steroidal anti-inflammatory drugs (NSAIDs) [2-4, 6]. We have presented here an extremely rare case of pill-induced esophageal injuries caused by ingesting excessive caffeine tablets that was diagnosed and followed up via endoscopy.

To the best of our knowledge, only three cases of esophageal injury caused by a caffeine overdose have been reported in the literature (Table 2). Iwamoto et al. reported on a patient who developed ulcer scars and a stricture encompassing the lower esophagus that persisted for 1 month [7]. Yanagisawa et al. also reported on a patient who had ingested 12 g of caffeine and experienced an esophageal stricture [8]. Finally, Nitta et al. described a man who consumed a caffeinated energy drink in which he dissolved 140 tablets of Estaron-mocha (14 g of caffeine) [9]. In that case, the patient ingested approximately 8 g of caffeine overall and his upper gastrointestinal endoscopy had shown circumferential ulcers in the lower esophagus without any gastroduodenal lesions. These patients consumed greater amounts of caffeine and presented with more serious clinical symptoms than the patient in this case. Therefore, it is likely that the amount of caffeine ingested may determine the severity of disease. However, Yasaka et al. described a man who consumed tablets containing 19.2 g of caffeine and was diagnosed with acute caffeine intoxication but a diagnostic endoscopy showed no lesions in the esophagus [10].

**Table 2:** Cases of pill-induced esophagitis caused by caffeine

Patient age (years), sex	Dose consumed	Symptoms	Endoscopic findings	Outcome	Reference#
Not noted	Not noted	Not noted	Circumferential ulcer scars and stricture in the lower esophagus	The stricture remained 1 month later	7
24, male	12 g	Nausea and vomiting	Severe stricture in the esophagus	Recovery after conservative therapy	8
19, male	8 g	Repeated vomiting	Circumferential ulcers in the lower esophagus without any gastroduodenal lesions	Recovery after conservative therapy	9
19, female	2.4 g	Epigastralgia, nausea, and a sore chest	Diffuse ulcers in the lower esophagus without any gastric ulcers or erosions.	Recovery after conservative therapy	Current case

Given the diversity of the esophageal findings among patients with acute caffeine intoxication, we suggest that multiple causative factors are involved in the severity of disease, such as the dose ingested, direct effects of caffeine on the esophageal mucosa, the form of the substance (i.e., tablet, powder, or liquid), and patient-related factors.

Many medications have topical toxic effects on the esophageal mucosa, including the production of local hyperosmolarity (as occurs with potassium chloride), pH and osmolarity-altering effects (as seen with ferrous sulfate), and intracellular uptake and cytotoxicity (as occurs with doxycycline and NSAIDs) [6, 11, 12]. However, the mechanisms of mucosal injury caused by some medications, including caffeine tablets, are unclear [13]. It is likely that the contents of tablets are sufficiently caustic to injure the esophagus if the tables are retained and dissolved therein. Theophylline, a metabolite of caffeine, occasionally induces gastroesophageal reflux when it is dissolved in the gastrointestinal tract. Shaikh et al. reported on a young man who presented with odynophagia and was diagnosed with theophylline-induced kissing ulcers in the middle esophagus [14]. Theophylline and caffeine, which are both methylxanthine derivatives, are structurally and toxicologically similar [15]. Thus, it is likely that the mechanism of esophageal injury from caffeine is similar to that of theophylline.

Patient-related risk factors of pill-induced esophagitis should be also taken into consideration. The most important patient-related risk factor is swallowing medicine with an inadequate volume of water. It is likely that the patient with suicidal thoughts in the present case may not have consumed enough water with these tablets. If so, the caffeine tablets may have lodged in the mid-lower esophagus and exerted a mucosal injury [16].

Although there were many reports from other countries of caffeine-related adverse effects caused by energy drinks or beverages to which powdered caffeine had been added [17-19], cases of ulcers and esophagitis resulting from the ingestion of caffeine in other forms have not been reported. Therefore, the liquid or powder form of caffeine is unlikely to be lodged in the esophagus, consequently resulting in a low risk of caffeine-induced esophagitis. Presumably, pills do not injure the esophagus directly if they pass rapidly through esophagus into the stomach. A previous report reported that 97 of 101 patients with acute caffeine intoxication in Japan had consumed caffeine in tablet form [1]. Therefore, patients in Japan are at a high risk of caffeine-induced acute esophageal damage.

Accordingly, we believe that multiple causative factors described above mutually contributed to prolonged contact between excessive caffeine tablets and the esophageal lumen, resulted in esophageal diffuse ulcers in this case [20].

In the present case, a diagnostic endoscopy was useful for determining the severity of esophagitis. In case of pill-induced esophagitis, the results from an endoscopy typically show small discrete regions of erosion or ulcers, including kissing ulcers, with adjacent normal mucosa in the mid-to-lower esophagus. Kim et al. investigated the endoscopic characteristics of pill-induced esophagitis and reported that kissing ulcers were seen in 34 cases (43.6%) among 78 patients examined [21]. In contrast, a diagnostic endoscopy in our case showed diffuse ulcers in the mid-to-lower esophagus, shallow circumferential ulcers in the middle esophagus, and non-circumferential ulcers with exudates in the lower esophagus. Therefore, the endoscopic features described here are atypical of pill-induced esophagitis. Presumably, most cases of pill-induced esophageal injuries involved a single tablet, or at the most a few tablets, and kissing ulcers, whereas the esophagitis was caused by a larger number of caffeine tablets in our case. Therefore, the caffeine tablets likely became lodged in the esophagus where they dissolved, thus leading to diffuse ulcers in the mid-lower esophagus. Differential diagnosis for diffuse ulcers includes reflux esophagitis, acute necrotizing esophagitis (ANE), and other cases of esophagitis. Severe reflux esophagitis may involve the entire circumference of the distal esophagus. However, reflux esophagitis was ruled out based on the absence of specific endoscopic findings, such as several linear, non-confluent red streaks extending up the esophagus; presence of hiatal hernia; and the complete recovery of the esophageal injury after 1 month [22]. ANE was also ruled out, because the endoscopic findings showed typical circumferential black lesions (referred to as “black esophagus”), while normal mucosa was seen in the lower esophagus in this case [23]. In addition, ANE usually arises in patients with severe conditions causing esophageal ischemia [23]. Endoscopic biopsies ruled out any underlying malignancy, viral or fungal infections, and eosinophilic esophagitis [22]. Until today, few reports have described caffeine-induced esophagitis that include endoscopic findings. Therefore, it is important that case reports be collated to improve information and knowledge on this condition.

Caffeine tablets are sold as over-the-counter supplements, and they are thought to be safe when consumed according to the manufacturers’ instructions (less than 500 mg/day) [24]. However, acute caffeine intoxication has been occurring more frequently than previously in Japan, as well as in other countries, due to excessive caffeine ingestion (2 g or more) [1, 24]. Plasma caffeine concentrations of more than 80–200 mg/L are considered lethal [24-26]. Supportive care is essential to treat caffeine intoxication, and some patients with an overdose of the substance may require numerous interventions, including hemodialysis [27]. The therapeutic approach to uncomplicated pill esophagitis is conservative. Proton pump inhibitors and histamine receptor antagonists are acceptable treatments for esophageal injuries, but cimetidine should be avoided because it may reduce the clearance of caffeine [28].

In conclusion, we have described a case of acute drug intoxication and diffuse ulcers in the mid-lower esophagus that were caused by ingesting excessive caffeine tablets. The number of patients with caffeine-induced esophagitis might be underestimated. To manage these cases appropriately, we should consider the risks associated with the induction of esophageal injury after excessive drugs have been consumed. In addition, taking a patient’s detailed medical history is necessary to understand the underlying pathophysiology correctly. If a patient presents with esophageal symptoms (such as retrosternal pain, odynophagia, and dysphagia) and is not in critical condition, an upper gastrointestinal endoscopy is advisable to properly assess any esophageal injury so that the best treatment to prevent severe gastrointestinal complications can be determined.
